# Early life stress induces sex-specific changes in cognitive and affective behavior associated with disrupted noradrenergic physiology

**DOI:** 10.1038/s41386-026-02463-6

**Published:** 2026-06-15

**Authors:** Savannah Brannan, Ben D. Richardson

**Affiliations:** Department of Pharmacology, Southern Illinois University—School of Medicine, Springfield, IL USA

**Keywords:** Stress and resilience, Neuronal physiology

## Abstract

Many psychiatric disorders are associated with specific risk factors, including biological sex and adversity in childhood, but the mechanisms underlying these relationships are unknown. Multiple cognitive and affective processes that are often disrupted in various psychiatric disorders are shaped by norepinephrine (NE) release throughout the brain, which is principally coordinated by noradrenergic locus coeruleus (LC) neurons with established sex differences in multiple properties, including stress sensitivity. Through application of a two-phase early life variable stress (ELVS) exposure to C57BL/6J mice to understand how early life adversity affects cognitive/affective behavior and LC physiology, we found that females displayed pronounced increases in novel environment exploration and reduced preference for sucrose. In addition, ELVS caused elevated activity in a familiar environment, modest deficits in Y-maze performance, and altered attention in an operant task in both sexes. A reduction in LC neuron sensitivity to corticotropin-releasing factor (CRF) and decreased excitability, partly due to an increase in action potential delay, was observed only in female mice exposed to ELVS, paralleling behavioral changes. CRF-induced changes in LC neuron activity were mediated by different preferential signaling pathways in naïve male and female mice - a potential mechanism for ELVS-induced sex-specific changes in LC physiology. Administration of the norepinephrine reuptake blocker reboxetine corrected ELVS-induced behavioral changes. Through this animal model of early life stress, we identified mechanisms involved in determining how stress and sex interact to cause LC activity dysregulation and related behavioral changes.

## Introduction

Adverse childhood experience (ACEs) exposure, often unpredictable, leads to increased risk for many neurodevelopmental and affective disorders, such as attention deficit hyperactivity disorder (ADHD), autism spectrum disorder, anxiety, and depression [1–8]. Although mature neurological systems are often resistant to acute stress, prolonged periods of stress in more malleable and immature systems can alter neurodevelopment [9–13]. Sex is a biological variable involved in determining the effects of threatening and non-threatening/deprivation ACEs, leading to adverse psychiatric outcomes, with females showing an increased risk for stress-related disorders in comparison to males [14–21]. Epidemiological studies indicate women may have increased stress-related disorder development risk/symptom exacerbation during life-phases characterized by hormonal changes that include puberty, menses, pregnancy, postpartum, and menopause [14–16].

Increased ACE exposures leading to attention, memory, affective, and/or mood disorders represent deficits in behavioral domains shaped by multiple circuits and modulatory neurotransmitters across the central nervous system. While there remain inconsistencies in reported data, the ACE category (abuse/threat, neglect/deprivation, household challenges/unpredictability) or type of stressor has been associated with particular changes in specific brain regions and associated behaviors [8, 22–25]. For example, some have linked deprivation (i.e., a lack of a nurturing environment or social stimulation) or neglect to reduced ventral striatal activity that parallels working memory and cognitive control (e.g., impulsivity, attention, ADHD) [24, 26, 27]. In contrast, exposure to threat or physical abuse in early development has been associated with increased alcohol abuse risk [28, 29], whereas non-threat ACE exposure is associated with depression [8]. Stress exposures used in animal models are not identical to human ACEs, but exposure to threatening stimuli (e.g., predator odor), deprivation (e.g., maternal separation) and/or unpredictable environments (e.g., variability of stressor exposure) affects comparable neurocircuits in animal models [30–35]. Resulting changes in circuits and behaviors in animals exposed to stress are often specific to certain stressor and timing combinations. Developing the links between stressor type and timing that affect specific circuits to negatively alter behavior is a key milestone in developing treatment approaches.

In considering the range of behaviors and processes linked to ACE exposure, many are housed across multiple circuits and also depend on optimal norepinephrine (NE) release from widely-projecting LC neurons: prefrontal cortex, hippocampus, amygdala, etc. [31, 36–43]. to modulate attention [44, 45], cognitive flexibility [46, 47], learned behavior [36, 42, 48, 49], anxiety [34, 42], reward [38, 42], and cerebellar-related processing [37, 50]. among others [43]. NE release during these behaviors or processes is highly dynamic, as appropriate tonic and phasic [44, 45, 51, 52] activity of LC neurons follow an inverted U-shaped function [43, 53, 54]. The firing activity of LC neurons is also sensitive to the central stress signaling peptide, corticotropin-releasing factor (CRF), which directly alters the activity of noradrenergic neurons in the LC [34, 55–58]. Animal model data indicate that, compared to males, female LC neurons have different basal physiological properties [59, 60], dendritic morphology [59, 61], cellular proteome [60], and elevated CRF sensitivity that would acutely increase basal NE release from LC neurons [55, 62, 63]. These sex differences in CRF-activated signaling in LC neurons may underpin the increased incidence of stress-related disorders in females [17, 64]. Despite the established role of NE in cognitive and affective behaviors and sex differences in LC and NE dynamics, how early stress interacts with the sexually dimorphic LC to shape behaviors associated with LC function (i.e., affect, cognition, and locomotion) is poorly understood. Therefore, we hypothesize that the noradrenergic locus coeruleus system (LC) may be a key neuromodulatory brain area that undergoes functional changes to drive stress- or ACE-related alterations in behavior.

Here, we apply a two-phase model [65] of stress exposure, combining maternal separation and early weaning with unpredictable stress at two early life time points, to model how ACE-like stressor timing and type interact with sex to shape affective, cognitive, and locomotor behavior. We then determined how exposure to this protocol affected LC physiology in a sex-specific manner. These results suggest that an interaction between sex and stress shapes affective and cognitive behaviors, in part, through alterations in the LC of C57BL/6J mice.

## Materials and methods

### Animals

Male and female C57BL/6J (B6J; Jackson Labs, #000664) or Dbh-tdTomato mice (described below) were purchased from Jackson Laboratory and/or bred in-house. To generate mice expressing tdTomato in locus coeruleus noradrenergic neurons under control of dopamine β-hydroxylase (Dbh-tdTomato mice), Dbh-Cre knock-in mice (B6.Cg-*Dbh*^*tm3.2(cre)Pjen*^*/J*; stock #033951) provided by Patrician Jensen, NIEHS [66] were crossed with homozygous Ai14 tdTomato reporter mice (B6.Cg-*Gt(ROSA)26Sor*^*tm14(CAG-tdTomato)Hze*^/J, Stock #007914) provided by Hongkui Zeng, Allen Institute for Brain Science [67]. All procedures involving animals were conducted in accordance with protocols approved by the Institutional Animal Care and Use Committee (#2022-100) of Southern Illinois University—School of Medicine.

### Early life variable stress (ELVS) animal model

**Fig. 1:**
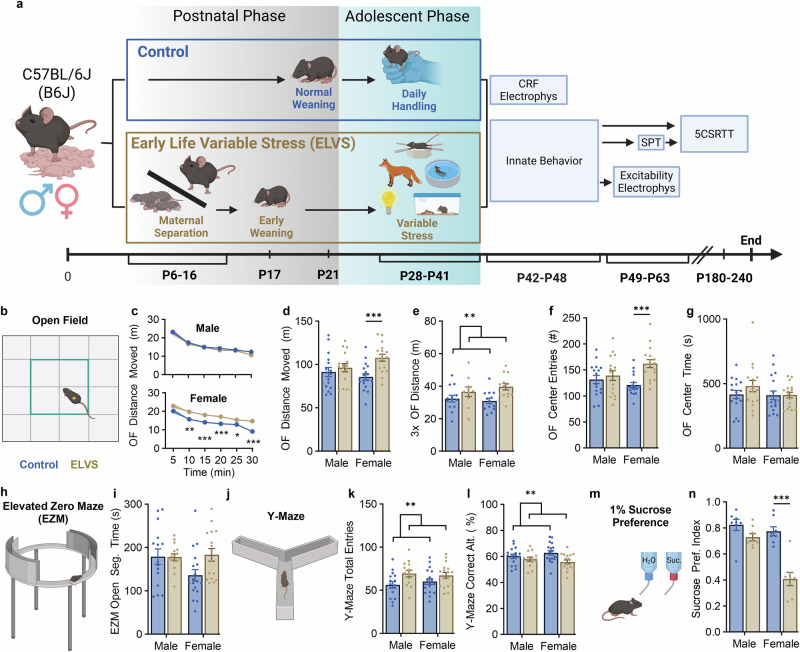
ELVS causes sex-independent and female-specific changes in cognitive and affective behavior. **a** Experimental timeline for all control and ELVS mice used in behavioral and electrophysiological assessments. **b–d** Total distance (meters, m) moved in the open field (OF) assay (**b**) across the entire 30 min session (**c**; males: *U* = 92.0–127.0, *p* = 0.18–0.99; females: *U* = 48.0–92.0, *p* = 0.00030–0.045) and in total (**d**; male: *t*(30) = 0.70, *p* = 0.49; female: *t*(33) = 4.08, *p* = 0.0003). **e** Total distance (m) moved in OF apparatus during the third exposure (3x OF) for 10 min (combined: *t*(47) = 3.13, *p* = 0.0030). **f,** **g** The number of entries into (**f**; male: *t*(30) = 0.63, *p* = 0.53; female: *t*(33) = 4.63, *p* < 0.0001) and total time spent (**g**) in the center of the OF arena. Time (seconds, s) spent (**i**) in the elevated zero maze (EZM, **h**) open arm. Y-Maze (**j**) total number of arm entries (**k**; combined: *t*(63) = 2.83, *p* = 0.0063) and correct arm alternation percentages (**l**; combined: *t*(63) = 2.94, *p* = 0.0046). Sucrose preference (**m**) index values as the volume of 1% sucrose/volume of all fluid consumed (**n**: male: *t*(14) = 1.89, *p* = 0.08; female: *t*(14) = 5.99, *p* < 0.0001). All results are presented as mean ± SEM for each group with circles representing data from individual mice. **b**–**l** N = 10–18 mice (6 weeks) per group. **n** *N* = 8 mice (9 weeks) per group. *p < 0.05, **p < 0.01, ***p < 0.001 with two-tailed unpaired t-test or Mann-Whitney U test for pairwise comparisons. **a**, **b**, **h**, **j**, **m** were created in BioRender.

Male and female B6J mice were subject to either a combined two-phase (postnatal and adolescent) early life variable stress (ELVS) paradigm or treated as controls with all mice in a litter (25 total litters) either exposed to ELVS or treated as controls (Fig. 1a) [65]. While there are a broad range of early life adversity paradigms applied to mice and rats [35], this paradigm was intended to mimic early life adversity types (neglect and threat) in a way that may lead to lasting rather than transient impacts. Specifically, the ELVS protocol was designed to address limitations of more standard early life adversity protocols that typically use only acute transient stressors of a single type. First, the timing of stress exposure was chosen to target early and late developmental sensitive periods. Second, repeated variable stressors were used to limit adaptation to each stressor and to engage circuits sensitive to major types of ACEs—neglect and threat. First, to produce a neglect-like stressor and also limit adaptation to maternal separation, mice in the ELVS group underwent maternal deprivation by being isolated together from dams for 4 h/day during postnatal day 6-10 (P6-10) with access to a warming pad, followed by early weaning at postnatal day 17 (P17). In the second phase (P28-41), each mouse was exposed to one stressor per day and each stressor was administered twice under close experimenter supervision starting with restraint by securing the feet to a metal rack with tape (20 min), forced ice-cold swim in a 40 × 10 cm filled container (3 min), 2,3,5-trimethyl-3-thiazoline (TMT) synthetic fox odor exposure (20 min), light cycle disruption by omission of one night of dark phase, and 48 h of social isolation. The order of stressors was the same for all mice. These stressors provided both threat and non-threat/deprivation forms of stress in an unpredictable order. Control and experimentally naïve (no behavioral assessment) male and female B6J mice were not separated from their dams, were weaned at P21, and were briefly handled 5 times per week for 10–15 min beginning at the same time as the stressors (P28) for the ELVS group.

### Behavioral tests

Innate behavioral tests not requiring prior water restriction or training were done from 6 to 7 weeks old during the animals’ active dark phase in a dimly lit room (~15–20 lux) with animals on a reverse light cycle. All behavioral assays, including open field, y-maze, elevated zero maze, novel object recognition and novel object placement, sucrose preference, rotarod, and five-choice serial reaction time task were performed as described previously [65] and in the Supplementary Information. Oral Reboxetine was provided in apple juice at 2 mg/kg 1 h prior to the performance of behavioral assays, where indicated.

### Ex vivo acute brain slice whole-cell electrophysiology

As performed previously [65] and in Supplementary Information, whole-cell patch-clamp recordings of putative and *Dbh* + (tdTomato) LC neurons were performed at 32 °C from visually identified LC neurons using the 4th ventricle as a landmark. Electrophysiological recordings, sampled at 20 kHz and filtered at 10 kHz, were acquired with patch pipettes filled with a K-gluconate-based solution and had a tip resistance of 5–7 MΩ. The calculated liquid junction potential of 15.5 mV. CRF responses were collected within 10 min following 500 nM CRF (human, rat, AnaSpec, Fremont, CA, USA) applied in the ACSF bath.

### Quantification and statistical analysis

**Fig. 2:**
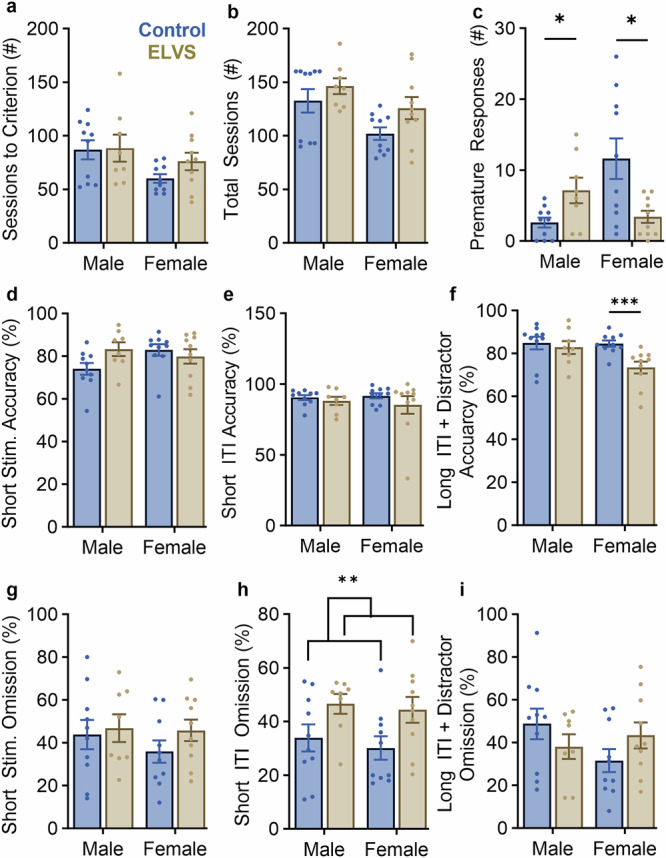
ELVS leads to sexually dimorphic changes in premature responses and female-specific distractibility in 5-choice serial reaction time task (5CSRTT). **a** Days needed to meet criteria during 5CSRTT training. **b** Days required to complete training and all session variation tests (male: *t*(16) = 0.98, *p* = 0.34; female: *t*(18) = 2.02, *p* 0.059). **c** Number of premature responses during the first training transition session in 5CSRTT training with reduced stimulus duration and intertrial interval (males: U = 15.0, *p* = 0.024; females: *U* = 22.0, *p* = 0.033). Trial accuracy % (**d**–**f**) and omission % (**g**–**i**) in test sessions with shortened stimulus duration in **d**, **g**, shortened intertrial interval in **e**, **h** (combined: *t*(36) = 2.97, *p* = 0.0053), and lengthened intertrial interval with a simultaneous sound distractor in **f** (male: *U* = 30.5, *p* = 0.42; female: *U* = 9.0, *p* = 0.0010), **i**.

Data were analyzed using two- or three-way ANOVA, repeated-measures ANOVA, or mixed-effects models, as appropriate to the experimental design; results of these factorial analyses are reported in Supplementary Table 1. Model assumptions were evaluated before pairwise testing. Normality of residuals was assessed using the Shapiro-Wilk test, and homogeneity of variance was evaluated using variance diagnostics. When assumptions of normality and homoscedasticity were met (*p* > 0.05), planned pairwise comparisons were conducted using paired t-tests for within-subject comparisons (drug effects), unpaired t-tests for between-group comparisons (stress effects), or Bonferroni post-hoc analyses as appropriate. When either assumption was violated (*p* < 0.05), nonparametric alternatives were used (Wilcoxon signed-rank tests for paired data and Mann-Whitney U tests for unpaired data). Pairwise comparisons were limited to pre-specified contrasts of interest (control vs stress), evaluated within sex when significant sex effects or interactions were present, and pooled across sex when only a main effect of stress was observed. Except for reboxetine treatment experiments, since only a single planned comparison was performed within each analysis, additional post hoc multiple-comparison adjustments were not applied. Statistical significance was defined as *p* < 0.05 for all main effects and pairwise comparisons. In order to limit Type II error in detecting complex stress-sex interactions, only for interactions with *p* < 0.10 were considered for pairwise comparisons, when accompanied by a significant main effect of stress (Figs. 1c and 2f). Data are presented as mean ± SEM, with individual data points representing individual animals in behavioral assays or individual neurons in cellular assays. Analyses and graphing were conducted in Clampfit 10.0 (Molecular Devices, San Jose, CA, USA), Easy Electrophysiology (Easy Electrophysiology Ltd, London, UK), Prism (10.0.2, Graphpad, Boston, MA, USA), and Igor Pro 8 (WaveMetrics, Lake Oswego, OR, USA).

## Results

### ELVS increases exploratory activity, impairs short-term memory, and induces anhedonia-like behavior

At 6-7 weeks of age, male and female control and ELVS mice were evaluated on a behavioral battery, including open field (OF), Y-maze, elevated zero maze (EZM), novel object recognition (NOR), and rotarod. Female ELVS mice displayed an increase in OF distance moved (Fig. 1b–d), which was absent in male and control female mice. To determine how exploration changed in a familiar environment, we evaluated the total distance moved during the second phase of the NOR task since this assay was performed in the same arena used in the open field assay, to which each mouse had been exposed two previous times. This measure of familiar environment exploration was significantly increased in ELVS-exposed mice (Fig. 1e), regardless of sex. Female ELVS mice also entered the center of the open field arena more frequently than control females (Fig. 1f), consistent with total OF locomotion. Mice of both sexes spent similar amounts of time in the center of the OF, regardless of ELVS exposure (Fig. 1g). Consistent with OF center time and absence of anxiety-like behavior, there were no significant differences between groups in the cumulative duration of time spent in the open arms of the elevated zero maze (Fig. 1h, i). In the Y-maze (Fig. 1j–l), ELVS-exposed mice of both sexes demonstrated increased arm entries (Fig. 1k) and decreased correct arm alternation sequences (Fig. 1l), indicating modestly impaired short-term memory relative to control mice. There were no significant sex- or stress-related differences observed in 24-h (h) (NOR, Supplementary Fig. 1a–d) or rotarod latency to fall (Supplementary Fig. 1e–g), which tested long-term (24 h) memory and motor function/coordination, respectively. At 9 weeks of age, female, but not male, ELVS mice displayed a significant reduction in sucrose preference (Fig. 1m, n) relative to control mice, suggesting ELVS induces an anhedonia-like state in female mice.

### ELVS causes sex-specific alterations in measures of impulsive-like behavior and attention

Additional separate cohorts of mice were water-restricted for the five-choice serial reaction time task (5CSRTT) training and testing, which was necessary to implement this behavioral task. There was no effect of ELVS on the time necessary for mice to reach the criterion threshold (>80% accuracy and <30% omitted trials; Fig. 2a). Although there were group effects of stress and sex on the total time to complete all training and testing sessions (Fig. 2b), there were no significant ELVS effects on these metrics for either sex. As one potential measure of impulsivity, the number of premature nosepoke responses for the first training trial when the stimulus duration and intertrial interval were shortened (Fig. 2c) was increased in males exposed to ELVS and decreased in ELVS-exposed females. Accuracy and trial omissions were similar across groups in most test sessions (Supplementary Fig. 2), including those with short stimulus durations (Fig. 2d, g). However, when the intertrial interval was reduced, ELVS-exposed mice of both sexes performed with similar accuracy (Fig. 2e) and omitted significantly more trials (Fig. 2h). 5CSRTT response accuracy was also reduced in ELVS female mice under challenging session parameters, including longer intertrial intervals in the presence of an audio distractor and varied target brightness (Fig. 2f), with no changes in omissions (Fig. 2i). These behavioral data suggest that ELVS exposure may lead to increased impulsive-like behavior in males while impairing attention capacity in females in challenging scenarios.

### Corticotropin-releasing factor (CRF) directly excites noradrenergic neurons based on ELVS exposure and via different signaling pathways in male and female mice

**Fig. 3:**
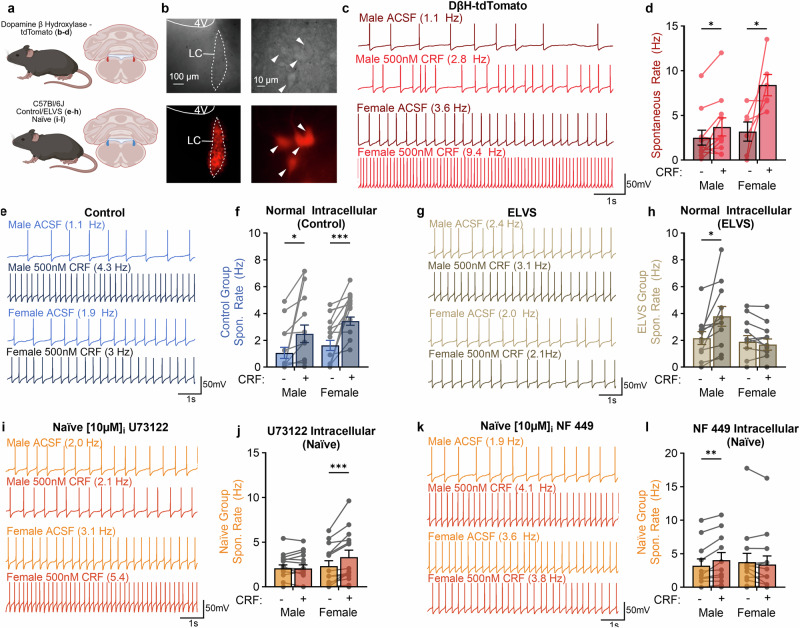
CRF excites LC neurons via different pathways in male and female mice and is lost in female mice after stress exposure. **a** Schematic of animals used and brain slice approach for recording form LC neurons in mice expressing tdTomato in dopamine β-hydroxylase (DβH)-expressing neurons, control, ELVS-exposed, or naïve mice. **b** DIC (top) and fluorescent tdTomato (bottom) image of the LC below the fourth ventricle (left) and a high magnification image of LC neurons in DβH-tdTomato mice. **c**, **e**, **g**, **i**, **k** Representative paired membrane voltage traces of spontaneous action potentials generated by LC neurons in ACSF and after bath application of 500 nM CRF from DβH-tdTomato (**c**), control B6J (**e**), and ELVS B6J (**g**) mice, or naïve mice with 10 µM U73122 (**i**) or 10 µM NF 449 (**k**) in the intracellular solution. **d**, **f**, **h**, **j**,** l** Group spontaneous action potential frequency of LC neurons in ACSF and after bath application of 500 nM CRF from DβH-tdTomato (**d**; male CRF: *t*(10) = 2.55, *p* = 0.029; female CRF: *t*(5) = 2.61; *p* = 0.048), control B6J (**f**; male CRF: *p* = 0.044; female CRF: *p* = 0.020), ELVS B6J mice (**h**; male CRF: *p* = 0.0015; female CRF: *p* = 0.99; female control CRF vs. ELVS CRF: *p* = 0.045), or naïve mice with 10 µM U73122 (**j**; male: *W* = 3, *p* = 0.93; female: *W* = -91, *p* = 0.0002) or 10 µM NF 449 (**l**; male: *t*(10) = 3.44, *p* = 0.0063; female: *t*(12) = 1.53; *p* = 0.15) in the intracellular solution. All group data are presented as mean ± SEM (bars) for each group, with connected circles representing paired pre- and post-CRF values from individual neurons. Two- (**d**, **j**, **l**) or three-way (**f**, **h**) RMANOVAs were used for group comparisons, followed by a paired t-test, Wilcoxon Signed-Rank test for single pairwise comparisons in two-way RMANOVAs or Bonferroni posthoc in three-way RMANOVAs to assess CRF effects. *n* = 6–11 neurons from *N* = 2–4 mice (6–8 weeks old) for (**c**, **d**), or *n* = 11–16 neurons from *N* = 3 mice (5–7 weeks old) for (**e**–**l**). *p < 0.05, **p < 0.01, ***p < 0.001 for pairwise comparisons. **a** was created in BioRender.

To understand how ELVS may impact CRF effects in the LC, we further assessed the degree to which CRF-evoked changes in LC excitability are dependent on sex and ELVS exposure. First, to determine the accuracy of targeting putative noradrenergic LC neurons recorded in wildtype B6J mice, we assess CRF (500 nM) effects on LC neuron activity using transgenic mice expressing tdTomato driven by noradrenergic marker, dopamine β-hydroxylase (Dbh-tdTomato mice) (Fig. 3a, b). Baseline and CRF-induced changes in firing behavior of tdTomato+ noradrenergic LC neurons were similar to baseline and CRF-induced changes in B6J mice of both sexes (Fig. 3c–f), with the exception of female Dbh-tdTomato mice that appear more sensitive to CRF. For naïve 5–6-week-old B6J mice, the same age mice were exposed to variable stressors, in the cell-attached configuration, CRF responses were similar to whole cell results (Supplementary Fig. 3a). There were also no significant changes in spontaneous firing rate when cells were held in the whole-cell configuration over a similar time period to that of CRF application (Supplementary Fig. 3b). Bath application of CRF (500 nM) onto LC neurons of male mice indicated that these neurons largely remained responsive to CRF in control (Fig. 3e, f) and ELVS (Fig. 3g, h), but there was a significant interaction between sex and stress on the effects of CRF on spontaneous firing. Specifically, in contrast to female control mice, LC neurons from ELVS female mice no longer responded by a change in firing rate (Fig. 3e–h) in response to bath-applied CRF.

Since it has been reported that female LC CRFR1 receptors predominantly couple to the G_s_ pathway [17, 55, 68], CRF activating CRFR1 coupled to G_s_, G_q_, and/or G_i_ pathways may underpin sex-specific ELVS-induced changes in physiology and behavior. In assessing G_q_ pathway dependence of CRF effects, inclusion of the phospholipase C (PLC) inhibitor 10 µM U73122 blocked the ability of 500 nM CRF to increase spontaneous firing of male noradrenergic neurons, but the sensitivity of female LC neurons to CRF remained (Fig. 3i, j). In contrast, the intracellular presence of 10 µM NF 449, a Gα_s_ inhibitor [69], to determine the dependence of G_s_-mediated signaling, blocked the ability of CRF to increase female LC neuron spontaneous firing, but some CRF sensitivity of male LC neurons remained partially intact (Fig. 3k, l). Acute CRF effects on evoked firing of LC neurons were limited in all conditions (Supplementary Fig. 4). Together, these data suggest that CRF preferentially signals through G_q_ signaling pathways in males and G_s_ signaling pathways in females.

### ELVS female LC neurons are less excitable after stress and show increased action potential delay attributed to A-type potassium channels

**Fig. 4:**
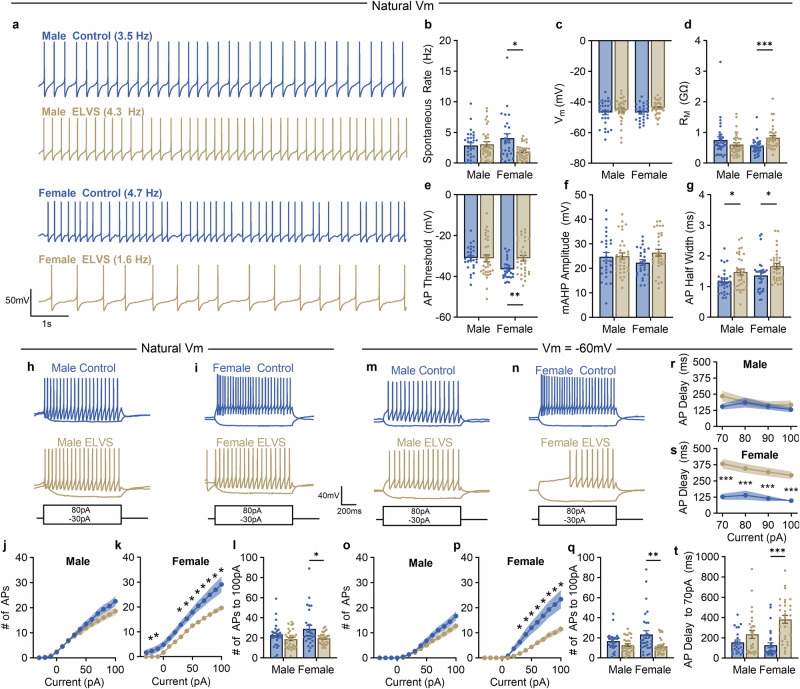
ELVS causes reduced LC neuron excitability and increased AP delay time in female mice. **a**,** h**,** i**,** m**,** n** Representative traces of male and female control and ELVS putative LC neurons when firing spontaneously (**a**), in response to +80/−30 pA current injection (1 s) when cells are at their natural resting membrane potential (**h,** **i**) or at −60 mV (**m**, **n**). Note that the same example recordings for both *V*_m_ states are provided from the same neuron, some of which are the same in both resting potential states because the natural V_m_ was at −60 mV. **b**–**g** Action potential spontaneous rate (**b**; males: *U* = 479.5, *p* = 0.82; females: *U* = 271.0, *p* = 0.014), resting membrane potential (**c**), membrane resistance (**d**; males: *U* = 443.0, *p* = 0.14; females: *U* = 253.5, *p* < 0.0001), action potential (AP) threshold (**e**; males: *U* = 410.0, *p* = 0.61; females: *U* = 256.0, *p* = 0.0069), medium after hyperpolarization (mAHP) amplitude (**f**), and AP half-width (**g**; males: *U* = 314.0, *p* = 0.020; females: *U* = 295.0, *p* = 0.023) for neurons from each group. **j**, **k**, **o**, **p** Number of action potentials elicited by putative LC neurons in response to 1 sec current injection steps from −30 to +100 pA for male (**j**, **o**) and female (**k**, **p**) mice with inter-step membrane potential varying naturally (**j**, **k**; females: *U* = 293.0–370.0, *p* = 0.00030–0.031) or maintained near −60 mV (**o**, **p**; females: *U* = 253.0–322.0, *p* = 0.0019–0.038). **l**,** q** Extracted from (**j**, **k**, **o**, **p**), number of action potentials elicited by putative LC neurons in response to maximum 100 pA for male and female mice with inter-step membrane potential varying naturally (**l**; males: *U* = 403.5, *p* = 0.11; females: *U* = 310.0, *p* = 0.025) or maintained near −60 mV (**q**; males: *U* = 284.0, *p* = 0.12; females: *U* = 253.0, *p* = 0.0019). **r**, **s** Delay time to first action potential after current injection onset present in putative LC neurons in response to 1 s current injection steps from +70 to +100 pA for male (**r**) and female (**s**; *U* = 118.0–148.5, *p* < 0.0001) mice with inter-step membrane potential maintained near −60 mV. **t** Extracted from (**r**, **s**), delay time to first action potential after current injection onset present in putative LC neurons in response to 70 pA current injection (1 s) for male (*U* = 258.0, *p* = 0.31) and female (*U* = 124.0, *p* < 0.0001) mice. All group data are presented as mean ± SEM (bars or shading) for each group, with circles representing data from individual neurons. Two- or three-way ANOVAs were used for group comparisons, followed by a t-test or Mann-Whitney U test for single pairwise comparisons to assess stress effects. *n* = 26–35 neurons from *N* = 4–5 mice (7–8 weeks old) for each group. For (**b**–**g,** **l,** **q,** **r**–**t**), *p < 0.05, **p < 0.01, ***p < 0.001 with two-tailed unpaired t-test or Mann-Whitney U test. For clarity in (**j**, **k**, **o**, **p**), only *p < 0.05 with Mann-Whitney U test for each current injection.

To address basal changes in LC neuron activity after stress, we first assessed spontaneous excitatory or inhibitory synaptic activity (Supplementary Fig. 5) using whole cell voltage-clamp recordings (Supplementary Information) from LC neurons in acute ex vivo brain slices and found no changes in either measure. Next, we assessed LC neuron excitability in ex vivo acute brain slices at 6 weeks of age (P37-44), immediately following stress, where no clear changes were observed in LC neuron excitability (Supplementary Fig. 6). Although one to 2 weeks after stress (7–9 weeks of age, P49-64), we found robust ELVS-induced female-specific changes in LC neuron activity (Fig. 4). Noradrenergic LC neurons from female ELVS mice, but not males, displayed reduced spontaneous firing rates, increased membrane resistance, and depolarized AP threshold when compared to controls (Fig. 4a, d, e), with no changes in resting membrane potential (*V*_m_; Fig. 4c). Both male and female ELVS LC neurons displayed modest, but significantly increased AP half-width (Fig. 4g). LC neurons from ELVS females also elicited fewer action potentials in response to current injection than from control females, a difference that was absent in males (Fig. 4h–l).

To account for variations in *V*_m_ across cells, we also assessed LC neuron evoked excitability with an initial resting potential of −60 mV set for all neurons, wherein ELVS females continued to show reduced evoked excitability (Fig. 4o–q). While absent in males (Fig. 4o), this excitability change appeared to be due, in part, to an increase in the delay time to the first action potential following current injection in females (Fig. 4s, t). Although the delay time to the first action potential was variable in all groups, only in female ELVS mice did almost all LC neurons display a notable delay (>40–50 ms; Fig. 4t).

To determine the mechanism involved in reducing excitability and increased action potential delay time in ELVS female LC neurons, we first investigated the role of ATP-sensitive K+ channels, which could account for changes in excitability, by removing both cyclic nucleotides (ATP and GTP) from the internal solution to promote maximal *K*_ATP_ activity and reduce variations in activity that could alter membrane resistance and *V*_m_ (Supplementary Fig. 7). Although removing ATP altered some stress effects on certain action potential properties, female ELVS LC neurons remained less excitable both at their resting potential (Supplementary Fig. 7i, k, l) and at −60 mV (Supplementary Fig. 7n, p, q), with a prolonged action potential delay time (Supplementary Fig. 7r–t).

**Fig. 5:**
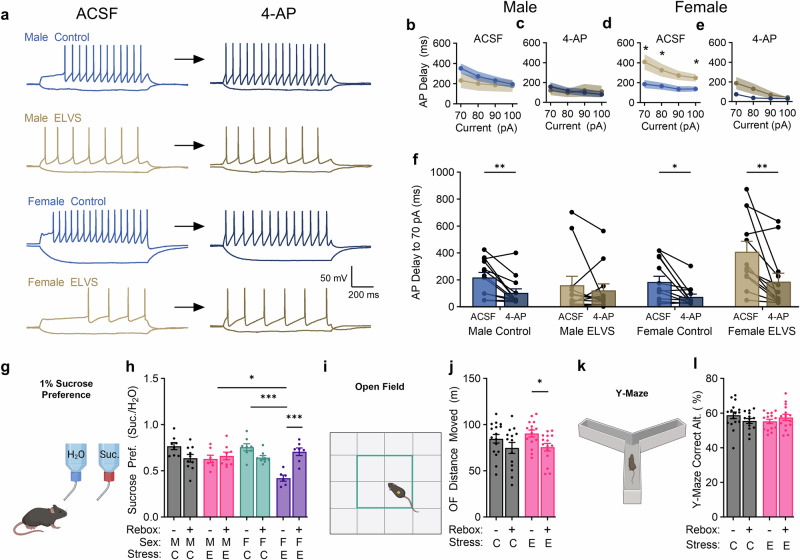
4-aminopyridine-sensitive A-type potassium channels determine AP delay time to reduce LC neuron activity, compensation of which with norepinephrine transporter inhibition restores ELVS-induced SPT and exploratory behavior. **a** Representative recordings from LC neurons in male (top) and female (bottom) control and ELVS mice before (left) and after (right) 5 min bath application of 1 mM 4-aminopyridine (4-AP). **b**–**e** Delay time to first action potential after current injection onset present in putative LC neurons in response to 1 s current injection steps from +70 to +100 pA before (**b**, **d**) and after 4-AP application (**c**, **e**) for male (**b**, **c**) and female (**d**, **e**; control: *U* = 40.0–45.5, *p* = 0.023–0.048; ELVS: *U* = 64.0–80.0, *p* = 0.47–0.84) control (blue) and ELVS (gold) mice with inter-step membrane potential maintained near −60 mV. **f** Delay time to first action potential after current injection onset present in putative LC neurons in response to 70 pA current injection (1 s) for male (control: *W* = 80, *p* = 0.0024; ELVS: *W* = 24, *p* = 0.38) and female (control: *W* = 79, *p* = 0.010; ELVS: *W* = 74, *p* = 0.0015) mice. **g–l** Performance of control (C, black, teal) and ELVS (E, pink, purple) male (M) and female (F) mice after oral consumption of vehicle (apple juice) or the NE transporter inhibitor reboxetine (2 mg/kg) in the sucrose preference (**g**, **h**; ELVS female vehicle vs. reboxetine: *p* = 0.0001; Control vs. ELVS female: *p* < 0.0001; ELVS male vs. female: *p* = 0.010), open field (**i**, **j**; control vehicle vs. reboxetine: *U* = 79.0, *p* = 0.18; ELVS vehicle vs. reboxetine: *U* = 52.0, *p* = 0.011), or y-maze (**k**, **l**) assays. All group data are presented as mean ± SEM (bars) for each group, with connected circles representing paired data from individual neurons (**f**) and circles representing data from individual animals (**h**, **j**, **l**). Three- or two-way ANOVAs were used for group comparisons, followed by a Bonferroni post hoc or Mann-Whitney U test for pairwise comparisons to assess effects of stress and/or oral reboxetine treatment on group performance. In (**a–f**), *n* = 11–14 neurons from *N* = 3 mice (7–8 weeks old) for each group. In **g–l**, sexes are presented separately in **h** and combined in (**j**, **l**) based on statistical results, *N* = 6–10 mice (7–9 weeks old) for each group and sex. *p* < 0.05, **p < 0.01, ***p < 0.001 for pairwise comparisons. **g**, **i**, **k** were created in BioRender.

Action potential delays and suppression of firing rate in response to depolarizing current may be due to activation of transient A-type voltage-gated potassium (Kv) channel-mediated currents (IA) present in LC neurons, which can be regulated by modulatory accessory proteins [70–72]. Therefore, we pharmacologically assessed the *I*_*A*_-dependence of this delay by bath applying 4-aminopyridine (4-AP, 1 mM) in recordings of evoked LC neuron firing (1 mM; Fig. 5a). In nearly all neurons from each group, 4-AP significantly reduced the action potential delay time (Fig. 5b–f), except for male ELVS mice, where the delay time was already short. In the presence of 4-AP, the action potential delay times were comparable in all groups (Fig. 5f), thereby indicating modulation of *I*_*A*_ by ELVS exposure to affect LC neuron physiology.

### Restoration of extracellular NE levels by reboxetine ameliorates ELVS-induced behavioral changes

Electrophysiological recordings of LC neurons indicate reduced excitability and increased delay to respond to current injection, both of which could lead to reduced distal NE release. Based on this premise, we determined whether ELVS-related behavioral changes could be restored by administration of reboxetine, a selective NE reuptake inhibitor. We found that oral reboxetine (2 mg/kg) restored female-specific effects of ELVS on sucrose preference behavior (Fig. 5g, h) and reduced exploratory behavior in ELVS mice (Fig. 5i, j), but did not alter y-maze performance (Fig. 5k, l). Overall, these data suggest that manipulating the LC system in a manner that is targeted based on the sex- and stress-dependent changes in physiology may be beneficial in restoring specific affective behavioral changes.

## Discussion

Here we find that mice exposed to ELVS exhibit specific behavioral alterations in exploratory locomotion in a familiar environment, short-term memory in early adulthood, and a reduced ability to maintain attention, regardless of sex (Figs. 1 and 2). In addition to alterations in impulsive-like behavior (premature responses) in male mice due to ELVS exposure, ELVS reduced premature operant responses (impulsive-like behavior), increased exploratory behavior, and an anhedonia-like state more robustly in female mice (Figs. 1 and 2). Interestingly, ELVS caused opposing effects in premature responses based on sex. This effect may, in part, depend on the lower premature responses in control males compared to control females, a theme reported elsewhere in animal models [73], but is less clear in humans [74, 75]. Changes in basal anxiety-like behaviors were absent due to ELVS exposure in the B6J mice used here, in contrast to BALB/C female mice [65] and the sex-independent effects of maternal separation on anxiety-like behavior [76].

LC neurons in ELVS-exposed female mice were less excitable, due in part to A-type Kv channel-mediated prolongation of the action potential delay time (Figs. 4 and 5). Unlike male BALB/C mice exposed to a similar ELVS protocol [65], properties of LC neurons from B6J male mice were remarkably similar to those from control mice. In addition to blunted CRF sensitivity specifically in female mice exposed to ELVS, in LC neurons from naïve mice, CRF-induced excitability changes were due to involvement of sex-specific signaling pathways (Fig. 3)—a possible explanation for sex-specific changes in basal LC neuron excitability due to stress exposure. Finally, pharmacological blockade of NE reuptake with reboxetine [77] restored a subset of ELVS-induced behavioral changes (Fig. 5g–l). While NE levels were not assessed directly to determine whether ELVS indeed led to reduced distal NE or whether reboxetine administration increased distal NE, our electrophysiology and behavioral data support this potential dynamic. Together, these data highlight the dynamics and importance of understanding the complex interplay between sex, age (critical periods), genotypic variation, stress exposure, and neuromodulatory circuit changes.

The unique impact of exposure to each stressor, their interactions, persistence of these behavioral changes into adulthood, and changes in behaviors related to coping [76], feeding [78, 79], or nociception [80, 81]—all shaped by LC noradrenergic function—are not addressed here. We aimed to determine the effects of early life ACE-like stressors on behavior and related noradrenergic physiology into late adolescence and early adulthood. Whether these changes continue to escalate or decline with age or are present in other stress-sensitive neurotransmitter systems [30, 82–84] is not addressed. In comparing behavioral and LC physiology changes in response to ELVS for B6J mice and BALB/C mice [65] to the effects of maternal separation in B6J mice alone [76], it seems that the timing and type of stress exposure indeed interacts with sex to drive the development of unique, and sometimes opposing, effects.

Since the LC noradrenergic system is also well-documented to be stress-sensitive [33, 34, 47, 76, 85–89], likely dependent upon CRF release from the central amygdala and paraventricular nucleus of the hypothalamus [32, 47, 55, 86, 90–92], CRF-induced changes in LC activity may affect multiple processes and behaviors known to be heavily shaped by LC neuron activity, including attention [93], cognitive flexibility [47], anxiety [31, 42], and memory [94]. The data presented here shed light on how the LC changes with multiple early life stress exposures and on the likely related increase in CRF release, which depends on sex. Specifically, ELVS sex-specifically alters tonic and evoked firing of LC neurons, both of which affect specific behavioral outcomes [44, 45, 52, 95]. While LC spontaneous and persistent firing rate changes observed in ELVS-exposed females will affect tonic firing, delays in current-evoked firing reduce the likelihood of generating an action potential in response to short depolarizing events - acting as a filter of fast synaptic inputs to reduce the likelihood of phasic firing and subsequent distal NE release. By disrupting both firing modes of LC neurons, these changes observed in females could result in altered NE signaling across multiple brain regions involved in attention, arousal, memory, and/or locomotion (i.e., prefrontal cortex, hippocampus, ventral tegmental area, and lateral cerebellar nucleus). We also note that changes in action potential delay time and general excitability more likely represent a shift in the distribution of these key properties across the population, rather than a change observed uniformly in all LC neurons. However, it is not clear how a change in these functional properties may relate to changes in specific molecularly-, anatomically- (target brain area), or functionally-defined LC neuron sub-populations.

These data broadly suggest that the treatment of stress- or specifically ACE-related disorders should consider sex and the timing of stress exposure as biological variables influencing outcomes. Females may be more sensitive to depression-like behaviors and ADHD-relevant deficits as a result of early life stress during comparable time periods used in this study. Individuals exposed to ACEs as adolescents may be particularly sensitive to developing negative neuropsychiatric outcomes later in life, based on sex differences in LC neuron physiology and central stress (CRF) sensitivity at this time. The specific sexually dimorphic molecular mechanisms underpinning LC physiology changes after exposure to the ELVS are still unclear, but they provide insight into key sex-specific mechanisms. Results from naïve mice (Fig. 3) establish that there are basal sex differences in how CRF exposure affects the firing of LC neurons, at least in the signaling pathways involved in CRF-induced increases in firing rate - preferential G_q_ signaling in males and G_s_ in females. Since these recordings were performed at an age comparable to that of mice during adolescent variable stress exposure, this sexual dimorphism in CRF signaling in the LC may contribute to sex-specific behavioral changes induced by ELVS. These data are consistent with other reports that female CRFR1s in the LC are predominantly G_s-_coupled and males have increased β-arrestin-related internalization of CRFR1 after acute stress [55]. Bridging the gap in future work to understand how two-phase early-life stress and acute stress affect CRF-dependent changes in LC physiology and related signaling, by sex, will be important for elucidating fundamental sex-specific processes that shape stress responses across both timescales. We predict that the predominant G_s_-dependent CRF actions in females, leading to PKA activation, will cause subsequent persistent changes in LC physiology (excitability, delay) in females that will be absent in males. In parallel, the predominance of *G*_q_ coupling could be protective in males due to its transient Ca^2+^-dependent action versus a prolonged upregulation of a signaling cascade.

This study may implicate the noradrenergic system, particularly the LC in the brain, as a region to explore biomarkers of dysfunction in female patients with a history of stress in early life. According to the DSM-V, cognitive issues are a core symptom of depression that include aspects of memory and attention being disrupted [96, 97]. These symptoms are in line with the female-specific behavioral impacts of a dual-phase stress model, pointing to increasing noradrenergic tone as a therapeutic avenue for treatment. Many drugs target this brain area, particularly second-line therapies for attention deficit hyperactivity disorder (ADHD), like reboxetine [98], which may prove to be effective in managing cognitive impairments in this patient group with depression and/or PTSD. This is relevant for many stress-related disorders, ranging from PTSD to substance abuse disorder that have sex differences in their prevalence ratios [64]. A more individualized understanding of how patients’ genetic and environmental risk factors interact with the type or timing of major stressful life events may increase the likelihood of therapeutic success when these variables are used to optimize drug choice.

## Supplementary information

Supplementary Information

Supplementary Table 1

## Data Availability

The datasets generated and/or analyzed during the current study are available from the corresponding author upon reasonable request. Source data for all figures, including behavioral assays and electrophysiology data, are provided in the Supplementary Information (Supplementary Table 1).
