# Supplementary material for: Early life stress induces sex-specific changes in cognitive and affective behavior associated with disrupted noradrenergic physiology
Source: Neuropsychopharmacology. 2026 Jun 15;51(9):1599–609. doi: 10.1038/s41386-026-02463-6 (PMC13389286; doi:10.1038/s41386-026-02463-6)
Supplement: Supplementary file 1 — Supplementary Information [file 41386_2026_2463_MOESM1_ESM.pdf]

Supplementary Information

Supplementary Materials and Methods

Early Life Variable Stress (ELVS) Animal Model

Male and female B6J mice were subject to either a combined two-phase (postnatal and adolescent) early life variable stress (ELVS) paradigm or treated as controls with all mice in a litter either exposed to ELVS or treated as controls (**Figure 1a**). ELVS was well tolerated and did not produce any obvious negative effects on the physical well-being of the mice in this group, with no loss of pups throughout or after exposure, changes in grooming/posture, or obvious differences in body composition. The ELVS paradigm consisted of two phases of manipulations, first from postnatal day 6-17 and the second from postnatal day 28-41. First, mice in the ELVS group underwent maternal deprivation by being isolated together from dams for 4 h/day during postnatal day 6-10 (P6-10) with access to a warming pad, followed by early at postnatal day 17 (P17). Pups were supplemented with nutrient gel (Nutra-gel, Bio-Serv, Flemington, NJ, USA) from P17 to P21. In the second phase, in order to maintain unpredictability of stressors to prevent adaptation, ELVS mice were exposed to sequential variable stressors from P28-41. During this variable stress phase, each mouse was exposed to one stressor per day and each stressor was administered twice under close experimenter supervision starting with restraint by securing the feet to a metal rack with tape (20 min), forced ice-cold swim in a 40 cm x 10 cm filled container (3 min), 2,3,5-Trimethyl-3-thiazoline (TMT) synthetic fox odor exposure (20 min), light cycle disruption by omission of one night of dark phase, and 48 hours of social isolation. The order of stressors was the same for all mice. These stressors provided both threat and non-threat/deprivation forms of stress in an unpredictable order. Control and experimentally naïve (no behavioral assessment) male and female B6J mice were not separated from dams, weaned at P21, and were briefly handled 5 times per week for 10-15 minutes beginning at the same time as the stressors (P28) for the ELVS group.

Behavioral Tests

Innate behavioral tests not requiring prior water restriction or training were done from 6-7 weeks old during the animals' active dark phase in a dimly lit room (~15-20 lux) with animals on a reverse light cycle. Mice were habituated to the testing room for 30 minutes prior to completion of behavioral testing. Mouse performance on all behavioral assays (**Figure 1**) was digitally recorded for analysis. Each apparatus was cleaned thoroughly with scent-free disinfectant between animals.

Open Field Test

Mice were placed in a white acrylic chamber (40 cm x 40 cm wide x 30 cm high square open top chamber) for 30 min with total distance, cumulative time in and number of entrances into center (20 cm x 20 cm) area relative to edges (10 cm border) were analyzed using the EthoVision XT 17 software (Noldus, Wageningen, the Netherlands).

Y-Maze

Mice were placed in a 3-armed "Y" shaped apparatus with symmetrical arms 35 cm in length, 30 cm wall height and 10 cm lane widths at 120-degree angles from one another. Total alternations, or the number of times the mouse enters 3 arms, were recorded and compared to correct (sequential) alternations, where every arm entered within the 3 entrances had not been entered in the immediate previous entrance.

Elevated Zero Maze

Mice were placed in the open arm of an elevated circular platform (50 cm diameter with 5 cm wide track) suspended 61 cm from the floor, with two opposing walled (20 cm high) segments and two open segments in between. The amount of time spent in and entries into the open versus closed segments were calculated using Ethovision XT 17.

Novel Object Recognition and Novel Object Placement Testing

The novel object recognition (NOR) assay was used to assess spatial memory. Following exposure to the open field for 30 min as the chamber acclimation period, the NOR task was

performed in the same open field chamber with two phases, each with a 10-minute duration. The first phase was the object habituation period, wherein two identical objects were placed equidistant apart on opposite sides of the chamber. In the second phase, one familiar object from the previous phase was replaced with a novel object at the same location. Each phase was done on separate days, with the novel object phase occurring 24 h after the same object phase. More time spent with the novel object, was interpreted as increased recall of the familiar object and related to memory and novelty seeking. The amount of time spent with the novel object versus the familiar object is calculated based on the time each mouse spent within 2 cm of the object using Ethovision XT 17. Because this assay was performed in the same arena used as the open field assay, the total distance covered during the second phase (novel object phase) of the assay was also used to determine exploratory activity in a familiar environment to which the mouse had been exposed two previous times.

Sucrose Preference Test

Starting at 7 weeks of age, after other behavioral testing and two weeks before sucrose preference testing (SPT), mice were progressively water-restricted, first to 4 hours of *ad libitum* water access per day for 2 days and then for 2 hours of access per day for 2 days, followed by two days of 24 hr access. This was repeated twice before limiting water access for 22 hrs prior to sucrose preference testing. For SPT, mice were placed in a clean empty cage for one hour with 2 bottles, one bottle containing 1% sucrose solution and a bottle containing water. The orientation (left vs. right) of the sucrose and water bottles were alternated between each mouse. The sucrose preference index was determined by comparing the amount of sucrose solution versus total fluid (1% sucrose + H_2O) consumed by each individual mouse.

Rotarod

Motor and vestibular function was evaluated using the accelerating rotarod (3.17 cm diameter, IITC life Sciences, Inc., Woodland Hills, CA, USA). This rod accelerated in rotations per minute (RPM) from 4 10 40 RPM during the span of 5 minutes. Each mouse received 3 trial

sessions per day for two consecutive days for a total of 6 trials. A 10 min rest period was given to the mouse between each trial. The time the mouse fell from the rotating rod onto the platform below was recorded.

Operant Five-Choice Serial Reaction Time Task (5CSRTT)

For 5SCRTT of attention and learning behaviors, separate control and ELVS cohorts of mice were trained using standard 5CSRTT protocols (1,2) and the apparatus was controlled using ABET software (Lafayette Instruments, Lafayette, IN, USA). Starting at 7 weeks of age, after other behavioral testing and two weeks before testing, mice were water-restricted initially to 4 hours of ad libitum access per day, reducing to 2 hours per day. A subset of these mice completed sucrose preference testing prior to initiation of 5CSRTT training. During training sessions, the mice were given sweetened condensed milk as a reward for correctly performing the task and weighed to ensure they were at the proper weight after water restriction. The operant conditioning chamber (30.5 x 24.1 x 29.2 cm) consisted of 2 Plexiglas sidewalls, an aluminum front wall and back wall, and a stainless-steel grid floor will be used to carry out this experiment. The front wall contained five nose poke apertures (2.5 x 2.2 x 2.2 cm each) with each aperture containing a light-emitting diode (LED) and an infrared sensor cable to detect mouse nose insertions. The mice were trained by progressively shortening the intertrial interval and stimulus duration for 30 minutes daily for 5-7 days per week to reach baseline performance with an intertrial interval (ITI) of 5 s and the stimulus duration of 0.8 s. Once mice reached the criterion threshold of 80% correct trials with <30% of trials omitted, they were assessed again on ten different variations with either shorter or longer ITI (**Figure 2**, **Supp. Figure 2**), shorter or longer stimulus duration, aperture light brightness, sound and light distractor manipulations, and combinations of these changes relative to the standard variables used during training. Response accuracy (% correct) and response omissions (# of premature responses during ITI) for each variable condition, along with the number of premature responses during specific training stages, were evaluated for differences based on mouse sex and treatment as control or exposure to ELVS.

Reboxetine Administration

In a separate cohort of 7-9-week-old control and ELVS mice, 2 mg/kg reboxetine diluted into apple juice or apple juice alone (100 µl) was given to control and ELVS B6J mice one hour prior to behavioral testing on SPT, open field, and Y-maze. To determine reboxetine effects on these behaviors, one behavior was evaluated each day on consecutive days with pre-test apple juice/reboxetine administered each day 1 h prior to each assay. SPT was completed first, followed open field and y-maze testing. Mice fully consumed 100 µl doses of either apple juice or 2mg/kg reboxetine in apple juice prior to task performance. The order of the behavioral assays was kept the same for all mice.

Ex vivo Acute Brian Slice Preparation

Either naïve, control, or ELVS-exposed B6J or DBH-tdTomato mice were used for electrophysiological recordings at the ages indicated for each experiment. While mice that completed behavioral testing were used for electrophysiology experiments, a limited cohort of mice was only used for electrophysiological assays. Mice that experienced water restriction (SPT, 5CSRTT) or reboxetine treatment were not used for electrophysiological recordings. All mice used for electrophysiology experiments were first anesthetized with 4% isoflurane, followed by intracardial perfusion with ice-cold oxygenated N-Methyl-D-glucamine (NMDG) artificial cerebrospinal fluid (NMDG-ACSF), which contained (in mM): 92 NMDG, 2.5 KCl, 0.5 $CaCl_2$, 10 $MgCl_2$, 1.2 NaH_2PO_4, 30 $NaHCO_3$, 20 HEPES, 25 D-glucose, 2 ascorbic acid, 2 thiourea, and 3 sodium pyruvate and had an osmolarity of 300–310 mOsm with pH adjusted to 7.3–7.4 with HCl. Coronal slices (250 µm thick) that included the LC were sectioned in ice-cold NMDG-ACSF using a Compresstome vibrating microtome (Processionary Instruments, LLC) and were transferred to a holding chamber containing NMDG-ACSF at 35 °C where the NaCl concentration increased steadily over 25 min. After 30 min, slices were transferred to a modified HEPES-based ACSF (HEPES-ACSF, 35 °C), which contained (in mM): 92 NaCl, 2.5 KCl, 2 $CaCl_2$, 2 $MgCl_2$, 1.2 NaH_2PO_4, 30 $NaHCO_3$, 20 HEPES, 25 D-glucose, 2 ascorbic acid, 2 thiourea, 3 sodium pyruvate,

and 3 myo-inositol and had an osmolarity of 300–310 mOsm with pH 7.3–7.4. After incubating slices in HEPES-ACSF at 35 °C for 1 hr, slices were maintained in HEPES-ACSF at room temperature until being transferred to the recording chamber where they were continuously perfused at a 3-5 ml/min with oxygenated ACSF, which contained (in mM): 125 NaCl, 2.5 KCl, 2 $CaCl_2$, 2 $MgCl_2$, 1NaH_2PO_4, 26 $NaHCO_3$, 20 D-glucose, 2 ascorbic acid, and 3 myo-inositol and had an osmolarity of 310–320 mOsm with pH 7.3–7.4.

Ex vivo Acute Brain Slice Whole-Cell Electrophysiology

Neurons in the LC were visualized using an upright microscope (Olympus BX51WI) with a 40x water-immersion objective, using infrared and differential interference contrast imaging with an infrared-sensitive camera and video monitor. Whole-cell patch-clamp recordings were performed at 32 °C maintained with an in-line solution heater from visually identified LC neurons using the 4th ventricle as a landmark. Noradrenergic neurons in the LC were identified by the presence of tdTomato in Dbh-tdTomato mice and action potential properties of putative noradrenergic LC neurons in B6J mice were verified to be similar to Dbh-tdTomato+ neurons.

Using a horizontal puller (P1000, Sutter Instruments, Novato, CA, USA), patch pipettes were pulled from filamented borosilicate glass capillaries (outer diameter 1.5 mm, inner diameter 0.86, Sutter Instruments), having a tip resistance of 5–7 MΩ when filled with potassium gluconate-based internal solution that contained (in mM): 139.6 mM K-gluconate, 0.4 mM KCl, 4 mM NaCl, 0.5 mM $CaCl_2$, 10 mM HEPES, 5 mM EGTA free acid, 4 mM Mg-ATP, 0.5 mM Na-GTP with an osmolality 285–290 mOsm and the pH adjusted to 7.2-7.3 with KOH (E_{Cl} = -85 mV). The calculated liquid junction potential of 15.5 mV was left uncorrected. For experiments assessing the role of ATP-sensitive potassium channels, all nucleotide salts were removed from the internal solution. For voltage-clamp recordings of synaptic activity, 5 mM QX-314 was included in the internal solution (iso-osmotically replaced NaCl) to block voltage-gated Na^+ channels. All signals were acquired at 20 kHz and low-pass filtered at 10 kHz via a MultiClamp 700B amplifier. Data were collected from the neurons with an input resistance >100 MΩ. If the series resistance for a

given recording was >30 MΩ or changed by more than 20%, the data were rejected for analysis. The current and voltage signals were recorded and digitized with a MultiClamp 700B amplifier and Digitdata 1440 controlled by Clampex 10 data acquisition software (Molecular Devices, San Jose, CA, USA). For 5-6 weeks old LC excitability and CRF response recordings, baseline excitability was collected 5 minutes after whole-cell and after evoked current injections (from -30 pA, increasing by 10 pA up to 100 pA with 10 seconds between each sweep) to ensure stable recordings and complete dialysis of the cytosolic space with intracellular solution. CRF responses were collected within 10 minutes following 500 nM CRF (human, rat, AnaSpec, Fremont, CA, USA) applied in the ACSF bath application. For recordings from 7-8-week old mice, spontaneous firing rates were collected first during a 3-5-minute free run, with sweeps excluded if they were 20% different from the average firing rate. Evoked firing responses to current injection steps were next performed using the same protocol as described above. In current-clamp mode, the neuronal membrane potential was kept at -60 mV by injecting an additional holding current, then the same current injection protocol was performed. The action potential threshold was determined by taking the average potential at which the dV/dt reached 20mV/ms when neurons fired spontaneously. To determine the resting membrane, an all-points histogram was generated for the spontaneous firing recording and the peak of the histogram (0.5 mV bins) was identified as the resting potential in highly active neurons. Neuronal membrane resistance was determined based on the holding current required for 5 mV step in voltage-clamp mode.

Supplementary References

1. Asinof SK, Paine TA (2014): The 5-choice serial reaction time task: a task of attention and impulse control for rodents. [no. 90]. *Journal of visualized experiments : JoVE* e51574.
2. Humby T, Wilkinson L, Dawson G (2005): Assaying Aspects of Attention and Impulse Control in Mice Using the 5-Choice Serial Reaction Time Task [no. 1]. *Current Protocols in Neuroscience* 31. https://doi.org/10.1002/0471142301.ns0805hs31

Supplementary Figure 1 – NOR/Rotarod

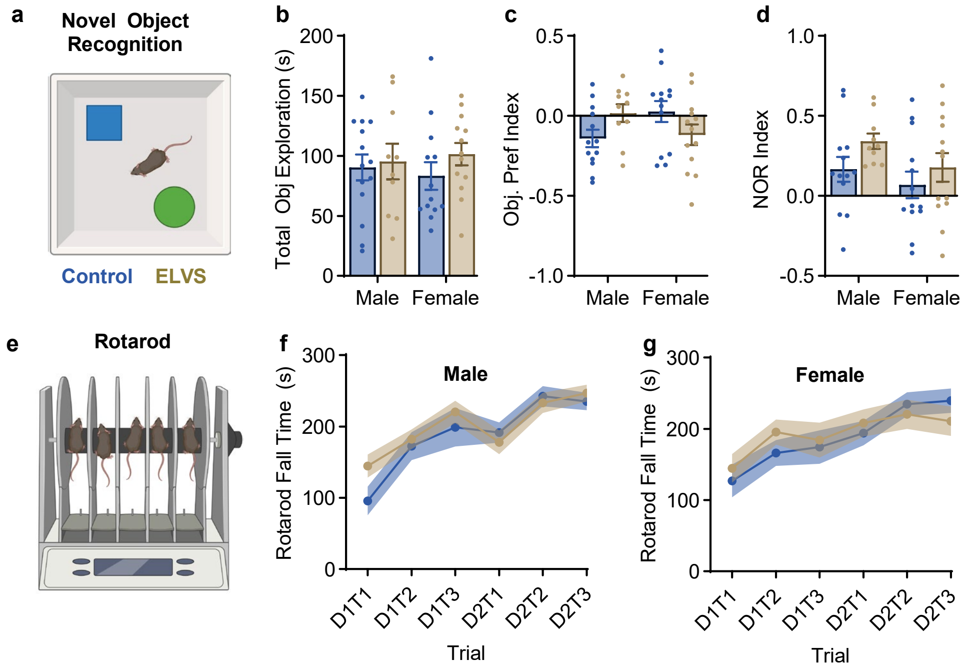

Supplementary Figure 1. ELVS exposure does not affect long-term (24hr) memory or motor function. (**a-d**) Novel Object Recognition (NOR) assay (**a**) total cumulative object exploration time (**b**), initial object preference index in the second phase (**c**), and NOR index in the third phase (**d**). (**e-g**) Two-day rotarod assay latency to fall times for each of three trials from both days in male (**f**) and female (**g**) control (blue) and ELVS (tan) mice. All results are presented as mean ± SEM for each group with circles representing data from individual mice. N = 9-14 mice (6 weeks) per group. Panels **a** and **e** were created in BioRender.

Supplementary Figure 2 – Full 5CSRTT

a

- Test 1: Long ITI
- Test 2: Short ITI
- Test 3: Reduced stimulus duration
- Test 4: Sound distractor (1st time)
- Test 5: Light intensity changes (random)
- Test 6: Sound distractor (2nd time)
- Test 7: Sound distractor + light intensity changes
- Test 8: Sound + light + long ITI
- Test 9: Sound + light + short ITI
- Test 10: Sound + light + reduced stimulus duration

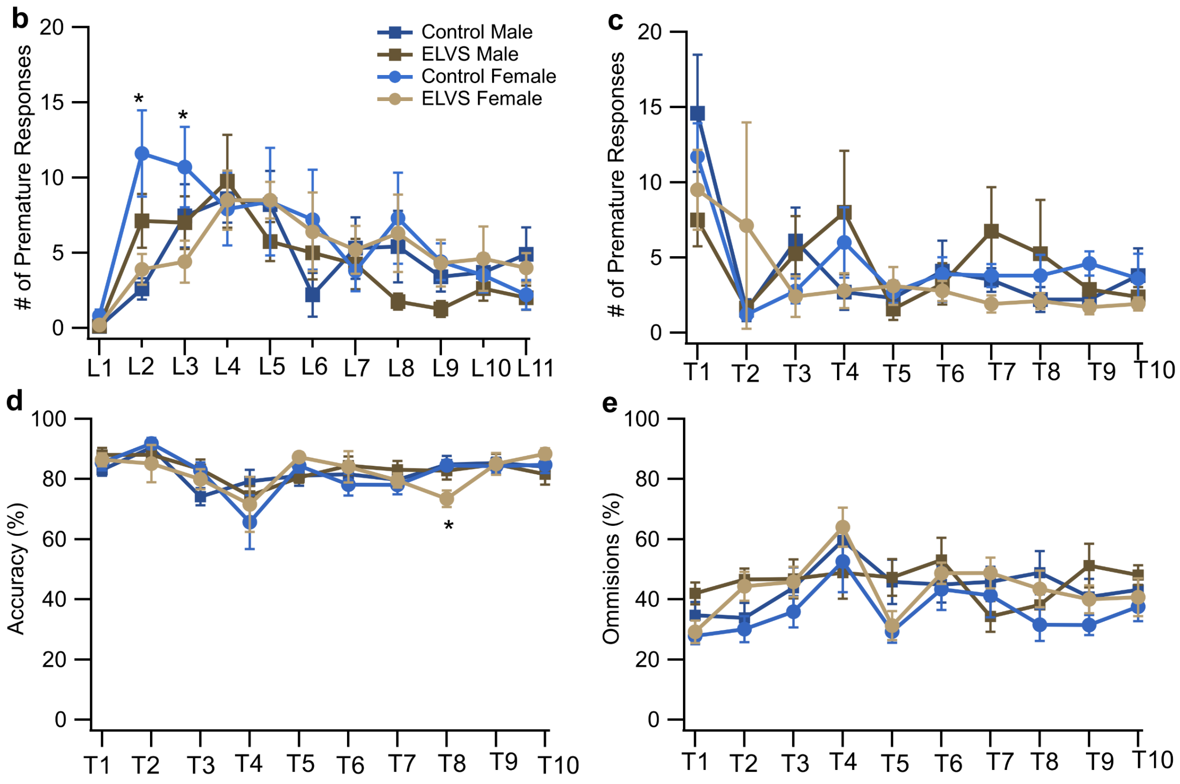

Supplementary Figure 2. 5SCRTT Premature Responses during training and accuracy and omissions during testing. (**a**) Listing of session variable changes for each of the ten test sessions. (**b**) Mean ± SEM number of premature trials of each group for the first time performing each training level. (**c-e**) Mean ± SEM number of premature responses (**c**), accuracy % (**d**), and omissions % (**e**) for each group during each test. N=7-10 mice (6-8 months old). * p<0.05 with unpaired two-tailed t-test for each sex for each session.

Supplementary Figure 3 – LCA & Holding Time

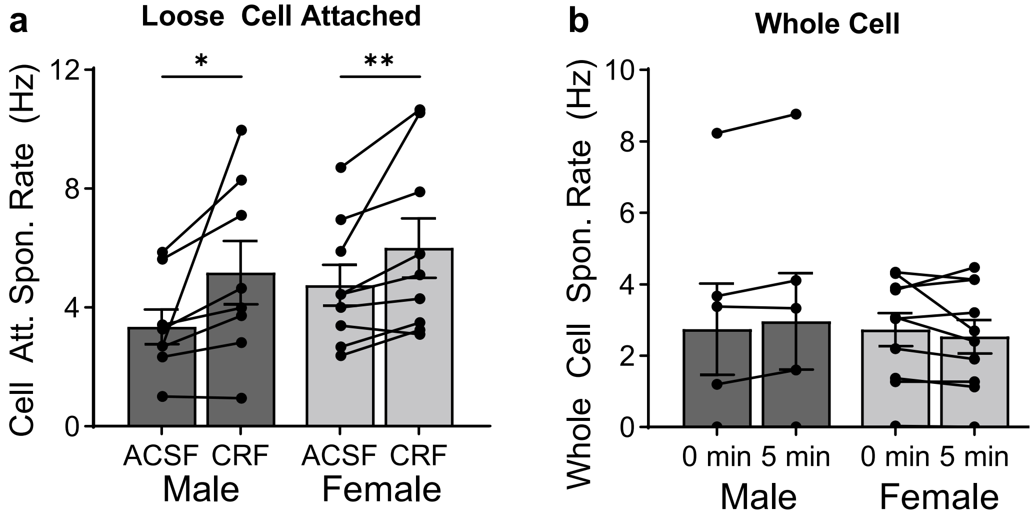

Supplementary Figure 3. Whole cell recording configuration and recording duration do not impact CRF-induced LC neuron excitability changes. (**a**) Spontaneous firing rate of putative LC neurons from 5–6-week-old male (W = -34.00, $p = 0.016$) and female (W = -43.00, $p = 0.0078$) mice in a loose cell attached configuration before and 5 min after bath applied 500 nM. (**b**) Spontaneous firing rate (Hz) of putative LC neurons from 5–6-week-old male and female mice recorded in the whole cell configuration at time = 0 min, beginning after a standard 5 min period after obtaining the whole cell configuration and after 5 min of recording. All group data are presented as mean ± SEM (bars or shading) for each group, with connected circles representing paired recording data from individual neurons. Two-way RMANOVAs were used for group comparisons, followed by a Wilcoxon Rank-Sum (paired) test (*$p < 0.05$, **$p < 0.01$) for single pairwise comparisons to assess CRF and time effects. n = 6-10 neurons from N=2-3 mice (5-6 weeks old) for each group.

Supplementary Figure 4 – CRF & Evoked Firing

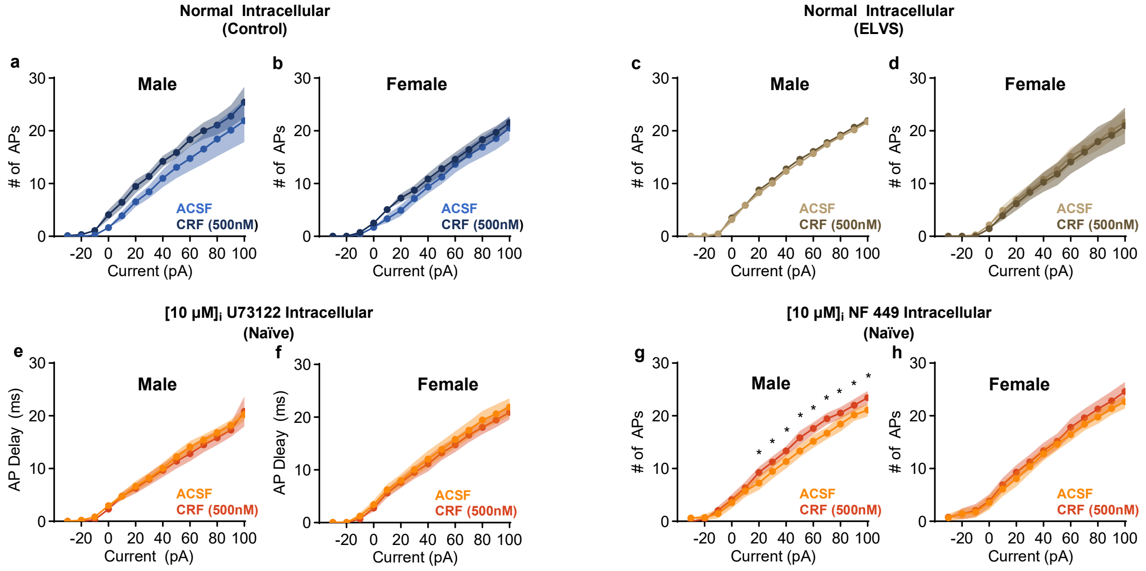

Supplementary Figure 4. CRF does not significantly impact evoked firing. (**a-d**) Number of action potentials elicited in response to 1 sec current injection steps (-30 to +100 pA) before and 5 min after bath application of CRF (500nM) by putative LC neurons from male (**a**, **c**) and female (**b**, **d**) control (**a**, **b**) and ELVS-exposed (**c**, **d**) mice. (**e-h**) Number of action potentials elicited in response to 1 sec current injection steps (-30 to +100 pA) before and 5 min after bath application of CRF (500nM) by putative LC neurons from naïve male (**e**, **g**) and female (**f**, **h**) naïve mice with either the phospholipase C inhibitor, U73122 (10μM; **e**, **f**; male: W = -30.00 – -66.00, *p* = 0.0010 – 0.039), or the Gα_s inhibitor, NF 449 (10μM; **g**, **h**), included in the intracellular recording solution. All group data are presented as mean ± SEM (shading) for each group. Two or three-way RMANOVAs were used for group comparisons, followed by a Wilcoxon Rank-Sum (paired) test (**p* < 0.05) for single pairwise comparisons to assess CRF effects within each group. n = 9-13 neurons from N = 3 mice (5-7 weeks old) for each group.

Supplementary Figure 5 – ELVS Synaptic Ephys

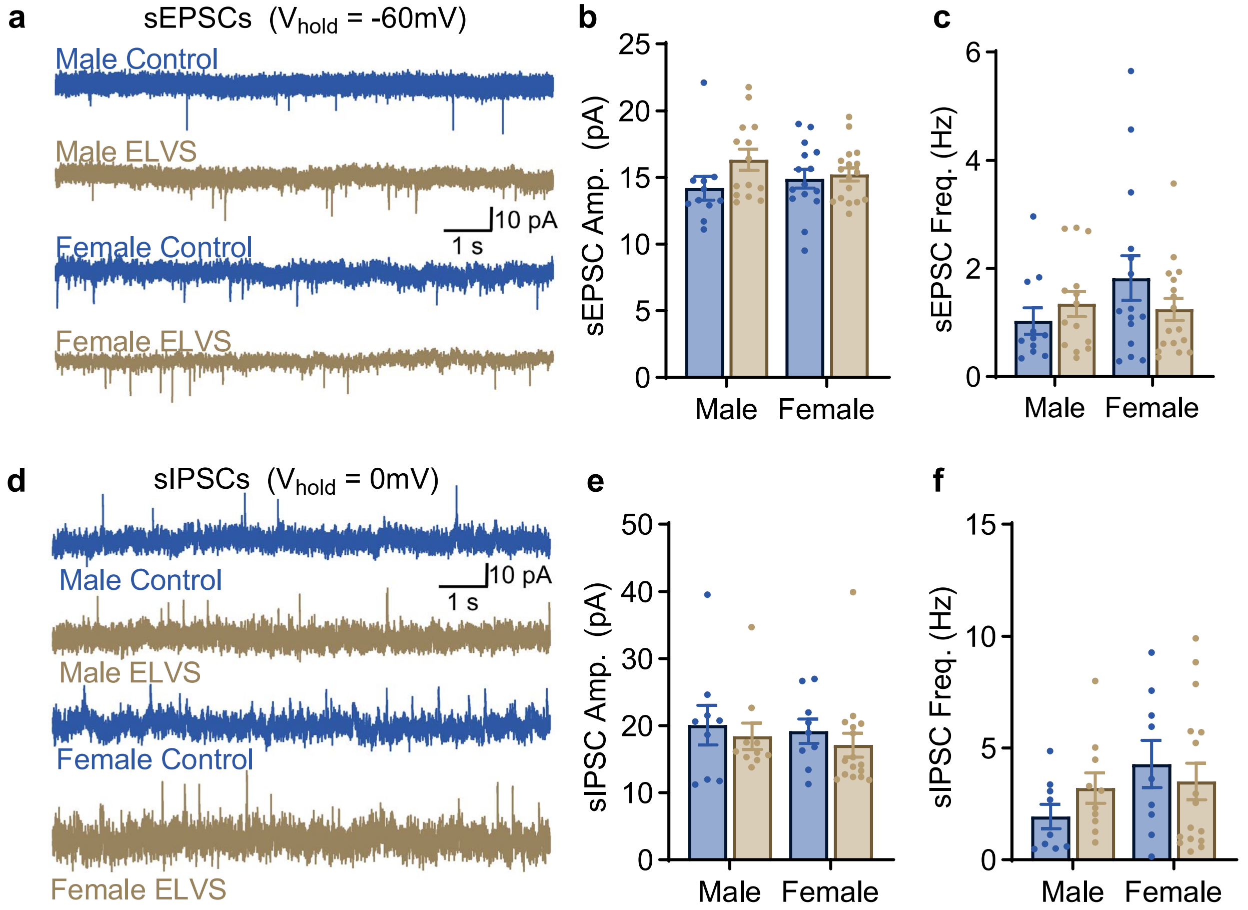

Supplementary Figure 5. ELVS does not impact LC synaptic physiology. (**a**, **d**) Representative voltage clamp recordings of male and female control and ELVS putative LC neuron spontaneous excitatory postsynaptic currents (sEPSCs; **a**; (V_{hold} = -60 mV; E_{Cl} = -85 mV) or spontaneous inhibitory postsynaptic currents (sEPSCs; **d**; V_{hold} = 0 mV; E_{Cl} = -85 mV). Group amplitude (**b**, **e**) and frequency (**c**, **f**) of sEPSCS (**b**, **c**) and sIPSCS (**e**, **f**). All group data are presented as mean ± SEM (bars) for each group, with circles representing data from individual neurons. Two-way ANOVAs were used for group comparisons. n = 9-17 neurons from N = 3-4 mice (7-9 weeks old) for each group.

Supplementary Figure 6 – No Ephys Change Early

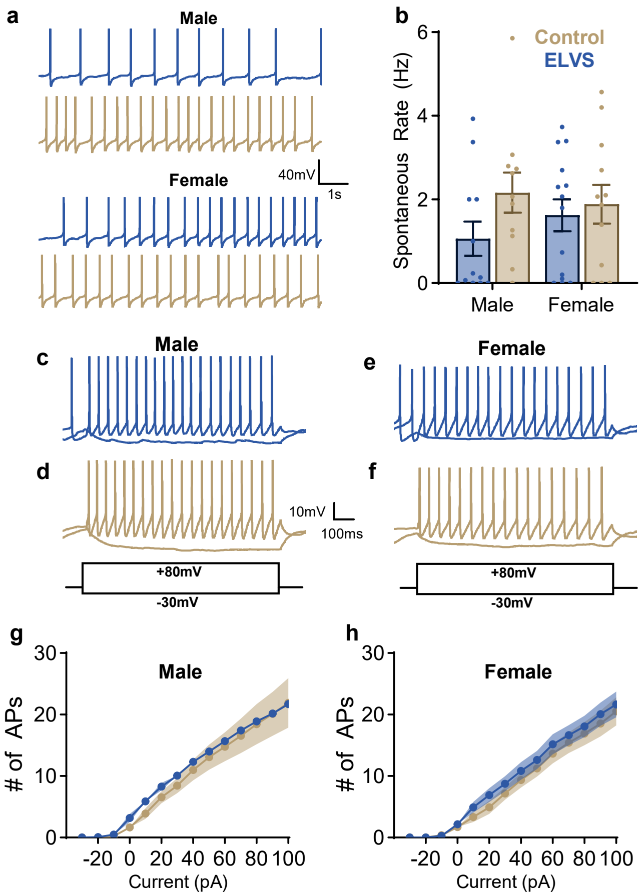

Supplementary Figure 6. No LC neuron excitability changes immediately after ELVS. (**a, c, d, e, f**) Representative recordings of the membrane potential from putative LC neurons obtained within one week after ending ELVS or control handling with neurons firing action potentials spontaneously (**a**) and from male (**c**, **d**) and female (**e**, **f**) control (**c**, **e**) and ELVS (**d**, **f**) mice in response to +80/-30 pA current injection (1 s) when cells are at their natural resting membrane potential. (**b**, **g**, **h**) Action potential spontaneous rate (**b**) and number of action potentials elicited by putative LC neurons in response to 1 sec current injection steps for male (**g**) and female (**h**) mice with inter-step membrane potential varying naturally. All group data are presented as mean ± SEM (bars or shading) for each group, with circles representing data from individual neurons. Two- or three-way ANOVAs were used for group comparisons. n = 9-13 neurons from N = 3 mice (6 weeks old) for each group. ANOVAs did not detect significant effects of stress exposure of sex in any cases.

Supplementary Figure 7 - No ATP/GTP

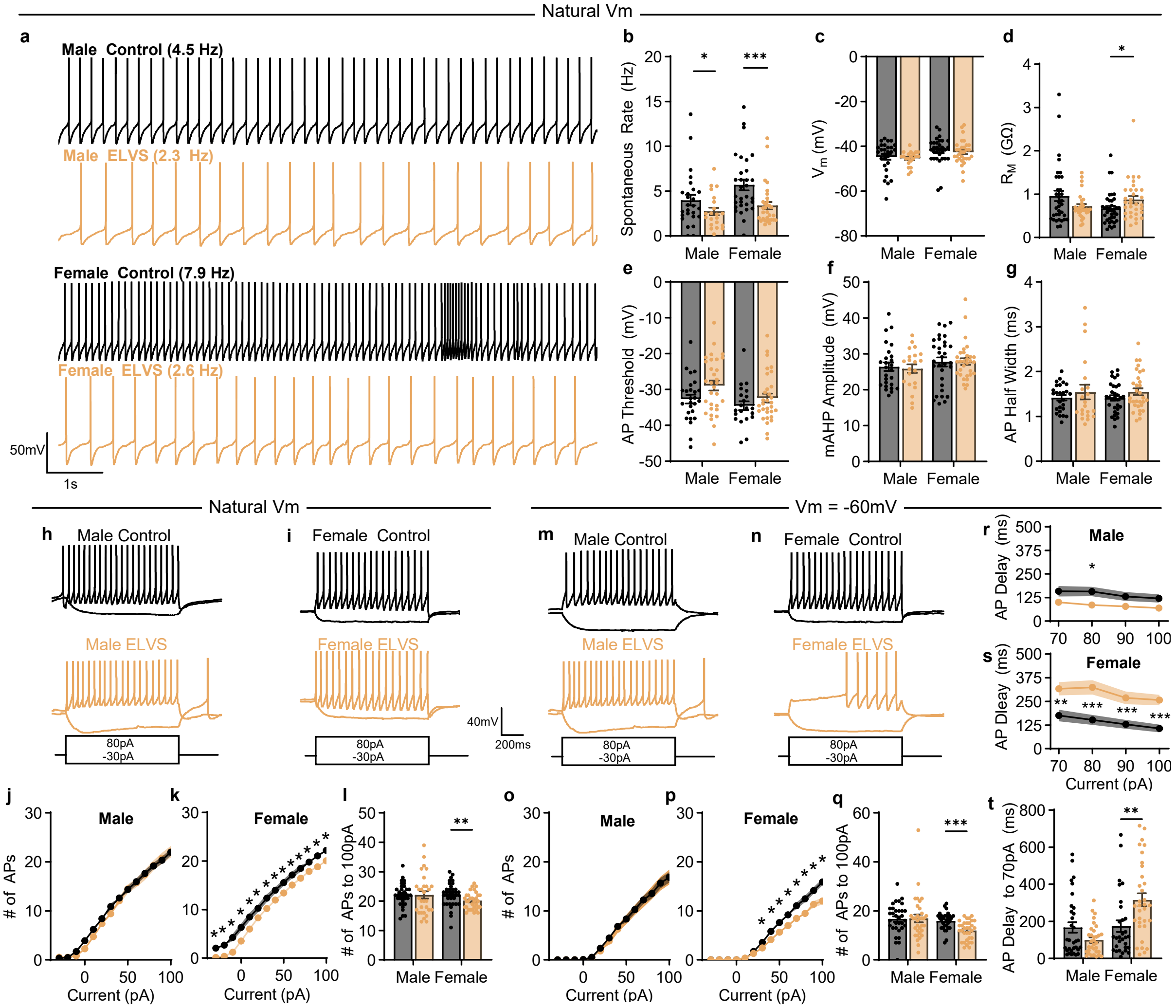

Supplementary Figure 7. ELVS-induced changes in LC excitability persist despite limiting K_{ATP} activity by removing intracellular ATP/GTP. All recordings completed with ATP and GTP omitted from the whole cell internal solution. (**a, h, i, m, n**) Representative traces of male and female control and ELVS putative LC neurons when firing spontaneously (**a**) in response to +80/-30 pA current injection (1 s) when cells are at their natural resting membrane potential (**h**, **i**) or at -60 mV (**m**, **n**). Note that the same example recordings for both Vm states are provided from the same neuron, some of which are the same in both resting potential states because the natural Vm was at -60 mV. (**b-g**) Action potential spontaneous rate (**b**; males: U = 174.0, *p* = 0.039;

females: U = 231.5, p=0.0007), resting membrane potential (**c**), membrane resistance (**d**; males: U = 460.5, p = 0.51; females: U = 401.5, p = 0.021), action potential (AP) threshold (**e**; males: U = 292.0, p = 0.077; females: U = 272.0, p = 0.33), medium after hyperpolarization (mAHP) amplitude (**f**), and AP half-width (**g**) for neurons from each group. (**j**, **k**, **o**, **p**) Number of action potentials elicited by putative LC neurons in response to 1 sec current injection steps from -30 to +100 pA for male (**j**, **o**) and female (**k**, **p**) mice with inter-step membrane potential varying naturally (**j**, **k**; females: U = 341.5 – 408.5, p = 0.0021 – 0.021) or maintained near -60 mV (**o**, **p;** females: U = 225.0 – 354.5, p = 0.00012 – 0.049). (**l, q**) Number of action potentials elicited by putative LC neurons in response to maximum 100 pA for male and female mice with inter-step membrane potential varying naturally (**l**; males: U = 460.0, p = 0.63; females: U = 367.0, p = 0.0061) or maintained near -60 mV (**q**; males: U = 475.5, p = 0.50; females: U = 225.0, p = 0.0001). (**r**, **s**) Delay time to first action potential after current injection onset present in putative LC neurons in response to 1 sec current injection steps from +70 to +100 pA for male (**r**; U = 337.0, p = 0.019) and female (**s**; U = 211.0 – 281.0, p = 0.0001 – 0.029) mice with inter-step membrane potential maintained near -60 mV. (**t**) Delay time to first action potential after current injection onset present in putative LC neurons in response to 70 pA current injection (1 sec) for male (U = 422.5, p = 0.23) and female (U = 281.0, p = 0.0029) mice. All group data are presented as mean ± SEM (bars or shading) for each group, with circles representing data from individual neurons. Two- or three-way ANOVAs were used for group comparisons, followed by a t-test or Mann-Whitney U test for single pairwise comparisons to assess stress effects. n = 20-37 neurons from N = 4-5 mice (7-8 weeks old) for each group. For **b-g**, **l**, **q**, **r-t**, *$p < 0.05$, **$p < 0.01$, ***$p < 0.001$, and for clarity in **j**, **k**, **o**, **p**, only *$p < 0.05$ with Mann-Whitney U test for each pairwise comparison.
